# Identifying metastatic ability of prostate cancer cell lines using native fluorescence spectroscopy and machine learning methods

**DOI:** 10.1038/s41598-021-81945-7

**Published:** 2021-01-26

**Authors:** Jianpeng Xue, Yang Pu, Jason Smith, Xin Gao, Chun Wang, Binlin Wu

**Affiliations:** 1 The Engineering Research Center of Synthetic Peptide Drug Discovery and Evaluation of Jiangsu Province, China Pharmaceutical University, No. 639 Longmian Avenue, Jiangning District, Nanjing, 211198 China; 2 Davinci Applied Technologies Inc, 476 Expressway Dr. S., Medford, NY 11763 USA; 3 Physics Department and CSCU Center for Nanotechnology, Southern Connecticut State University, New Haven, CT 06515 USA; 4 Department of Biomedical Engineering, Rensselaer Polytechnic Institute, Troy, NY 12180 USA; 5 Natural Sciences Department, LaGuardia Community College, City University of New York, Long Island City, NY 11101 USA; 6 Center for Optics Research and Engineering of Shandong University, Jinan, 211198 China

**Keywords:** Optical techniques, Optical spectroscopy, Fluorescence spectroscopy, Optics and photonics, Cancer, Cancer screening, Metastasis

## Abstract

Metastasis is the leading cause of mortalities in cancer patients due to the spreading of cancer cells to various organs. Detecting cancer and identifying its metastatic potential at the early stage is important. This may be achieved based on the quantification of the key biomolecular components within tissues and cells using recent optical spectroscopic techniques. The aim of this study was to develop a noninvasive label-free optical biopsy technique to retrieve the characteristic molecular information for detecting different metastatic potentials of prostate cancer cells. Herein we report using native fluorescence (NFL) spectroscopy along with machine learning (ML) to differentiate prostate cancer cells with different metastatic abilities. The ML algorithms including principal component analysis (PCA) and nonnegative matrix factorization (NMF) were used for dimension reduction and feature detection. The characteristic component spectra were used to identify the key biomolecules that are correlated with metastatic potentials. The relative concentrations of the molecular spectral components were retrieved and used to classify the cancer cells with different metastatic potentials. A multi-class classification was performed using support vector machines (SVMs). The NFL spectral data were collected from three prostate cancer cell lines with different levels of metastatic potentials. The key biomolecules in the prostate cancer cells were identified to be tryptophan, reduced nicotinamide adenine dinucleotide (NADH) and hypothetically lactate as well. The cancer cells with different metastatic potentials were classified with high accuracy using the relative concentrations of the key molecular components. The results suggest that the changes in the relative concentrations of these key fluorophores retrieved from NFL spectra may present potential criteria for detecting prostate cancer cells of different metastatic abilities.

## Introduction

The ability to metastasize is a fateful characteristic of certain malignant tumors, which currently account for the majority of cancer-related deaths. One third of all people worldwide will be diagnosed with a form of cancer during their lifespan. Currently, one third of these diagnosed cases will result in death due to metastasis. However, only a few notable methods have been developed to measure the metastatic ability present in a variety of cancers. In particular, prostate cancer afflicts more men than any other form in the western world—ranking third in terms of mortality after lung cancer and colorectal cancer^1^. The detection of prostate cancer at an early stage is paramount for improving patient prognosis, as prostate cancer detected early has a significantly higher chance of successful treatment. One promising non-invasive method to diagnose cancers without removing tissue is based on optical spectroscopy, which was shown to be able to determine the state of tissue ex vivo^2–5^ as well as in vivo^6–8^ in previous studies. To characterize the properties of normal, benign, and malignant tissue and cells, one major focus in optical biopsy is measuring native fluorescence (NFL) spectra, which plays an important role in discrimination of cancerous tissue from normal tissue since the early studies of Alfano in 1980s^2^.

It is widely acknowledged that the emission spectrum of a tissue/cell is a superposition of spectra of various salient fluorophores^9–11^. The main building-block fluorophores present in tissue include tryptophan, reduced nicotinamide adenine dinucleotide and its phosphorylated form [NAD(P)H], flavin adenine dinucleotide (FAD), collagen, and elastin. These molecules appear with different amounts and structure in tumor evolution and these changes can be revealed by NFL spectra^2–5^.

Tryptophan is an essential amino acid. It cannot be produced by cells and must be supplied in the diet. Tryptophan is needed for protein synthesis. It is transported via large amino acid transporter system (LAT1/CD98) into the cells, where it is degraded to kynurenine by the enzyme indoleamine-2,3-dioxygenase^12,13^. Many studies have shown that the degradation of tryptophan is a mechanism that tumors select to achieve immune escape^12–15^. The failure of immune system control on the growth of tumor cells leads to the fast development of a tumor^12,13,16^. Since there are more large amino acid transporters on the cell membrane of aggressive cancer cells, tryptophan can be taken up more efficiently in such cells than others from the surrounding environment.

Tryptophan is required for T lymphocyte effector functions^17,18^. The T cells in immune system are particularly susceptible to low tryptophan concentrations, which results in energy and apoptosis so that cancer cells can escape from the immune detection and survive^12,13^. Moreover, a pair of receptor and ligand proteins, PD-1 receptor on T cells and PD-L1/2 on cancer cells, have been observed to permit cancer cells to escape the immune system when PD-1 receptor is activated in low tryptophan environment. When the PD-1 binds to PD-L1/2, it suppresses the T cell activity, causing T cell apoptosis. These interactions help cancer cells escape from the immune detection and develop toward increasingly aggressive forms. Therefore, direct monitoring of the tryptophan level in cells/tissue can be used to investigate the immune escaping ability of the cancer cells and the metastasis ability of prostate and other cancer cells with low, mild and high aggressiveness.

We use NADH to denote both NADH and NADPH, since (a) the emission spectra from NADH and NADPH are identical^19^, and (b) the cellular content and fluorescence quantum yield of NADH are higher than that of NADPH, therefore we expect that our results mostly indicated the concentration of NADH^20^. NADH is the principal electron donor in cellular metabolism. The nicotinamide adenine dinucleotide (NAD^+^), a natural coenzyme, regulates immune responses and creates homeostasis via a novel signaling pathway^13,21^. Interestingly, quinolinic acid, which is a neurotoxic catabolite of the kynurenine pathway, is the major pathway for the de novo NAD^+^ synthesis. More importantly, the immunoregulatory properties of NAD^+^ are strongly related to the overexpression of tryptophan hydroxylase 1 (Tph1).

Quantification of the key biomolecular components within tissues/cells may present potential criteria for cancer detection and classification. Since the fluorescence signal is a mixed signal due to multiple fluorophores and the spectrum possesses high dimensional data, it is important to unmix the NFL spectra and reduce the dimension so that key components in the signal can be retrieved and analyzed. The measurement of the oxidation state of pyridine nucleotide [NAD(P)H] and the relative concentrations of other fluorophores is possible by decomposing the cellular spectral signal into individual fluorophores^22^. To unmix the NFL data, and retrieve the spectra and relative concentrations of the components of our interest, algorithms such as principal component analysis (PCA)^23–25^ and nonnegative matrix factorization (NMF)^5,24,26^ have been used.

In this study, the native fluorescence spectra of low metastatic (LNCaP), moderately metastatic (DU145), and advanced metastatic (PC-3) human prostate cell lines^27,28^ were studied using the selected excitation wavelength of 300 nm to investigate the key molecules such as tryptophan and NADH.

The basis spectra of these key fluorophores were obtained along with their relative concentrations, which were subsequently used to classify the samples using support vector machines (SVMs)^29^. Our study provides a possible diagnosis method based on the changes of relative concentrations of tryptophan and NADH in cells which indicate the metastasis competence and the risk levels of cancer in patients.

This study was focused on the classification of prostate cell lines with different metastatic ability based on NFL and machine learning. The purpose was to detect invasiveness (metastatic potential) of prostate cancer cell lines, and find a quantitative relationship between the metastatic potential and the molecular information revealed by fluorescence spectroscopy. We first used PCA for linear unmixing. The component spectra and the relative concentrations retrieved by PCA may include negative values and cannot be attributed to particular fluorophores. Besides PCA, we also used NMF, which can potentially retrieve components for individual fluorophores due to the nonnegativity constraint.

## Samples and methods

### Sample preparation and cell lines

DU145 (ATCC, Virginia) cells were cultured in DMEM medium (Sigma, Missouri), supplemented with 10% fetal bovine serum (FBS) (Thermo Scientific, Massachusetts), 25 units/mL penicillin, and 25 mg/mL streptomycin (Sigma, Missouri). The LNCaP (ATCC, Virginia) cells were cultured in RPMI 1640 medium with 2 mM l-glutamine and 10% FBS. The PC-3 (ATCC, Virginia) cells were cultured in F-12K Medium (Sigma, Missouri) with 10% FBS. All cell lines were incubated under 5% CO_2_ atmosphere at 37 °C. The cells were harvested at more than 95% cell confluence. The 0.25% trypsin-ethylenediaminetetraacetic acid (EDTA) (Sigma, Missouri) were used to treat the cell for less than 1 min. Then 5 mL cell culture media were used to wash the cells away from the bottom of the flask. The solutions were centrifuged at 2000 revolutions per minute (rpm) for 5 min. The supernatant with remaining trypsin were aspirated. The cells were resuspended with 5 mL phosphate buffered saline (PBS, Sigma, Missouri), and centrifuged again. The supernatant (almost all the solution) was removed. The remaining centrifuged cells were resuspended to ∼3.6 × 10^6^ cells/mL with PBS. The cells were then transferred to a quartz cuvette (NSG Precision Cells, New York) with inner dimension of 1 cm × 1 cm × 4 cm for the subsequent fluorescence experiments. The cell suspensions were vortexed to mix evenly before each measurement. After the experiments, the rate of living cells was estimated using trypan blue solution (Sigma, Missouri).

### Experimental parameters and data acquisition

In this study, a Fluorolog-3 spectrofluorometer system (HORIBA Scientific, Piscataway, New Jersey) was used to measure the NFL spectra of the cells. The excitation light was set to be 300 nm with 0.5 μW power deposition and 5 nm spectral slit, and used to shine on samples with a spot size of ~ 3 mm × 1 mm. The scan speed was set to be 300 nm per minute with each scan taking less than 1 min. The fluorescence signal with a spectral resolution of ~ 1 nm was collected in the range of 320 nm and 580 nm. To examine the amount of the background fluorescence, the emission spectrum of the supernatant from cell samples was also measured in the quartz cuvette. The intensity of the fluorescence signal at 340 nm measured from the supernatant was only 0.1 to 1.5% of that collected from cell samples. This background spectrum was deducted from the spectra for the cell samples before subsequent analysis. The excitation wavelength of 300 nm was selected because it was shown to be an optimal wavelength to collect both tryptophan and NADH fluorescence signals^30^. Especially, tryptophan showed a quantum yield (QY) of 0.2 to 0.35, higher than the other fluorophores in cells and tissue^30^.

### Analytic methodology

The spectral data were first analyzed using PCA and NMF to unmix the fluorescence signals to reduce data dimensionality and acquire valuable features. PCA is a matrix decomposition technique which finds the uncorrelated orthogonal components (principal components or PCs) which account for the largest variances in the data. Usually, the PCs do not directly correspond to the basis spectra of the key fluorophores, but are instead linear combinations of them. The PCs are found as eigenvectors for an eigenvalue equation of the covariance matrix of the spectral data matrix. In the matrix form, *X*^*m*xn^ = *W*^*m*x*r*^ × *H*^*r*x*n*^, where *X* is the input data matrix of dimension *m* wavelength values by *n* spectra, *W* is the matrix containing *r* basis spectra (PC loadings), and *H* is the corresponding weight matrix (PC scores). In practice, the eigenvectors can be found by singular value decomposition (SVD) of mean-centered data matrix *X’*. In matrix form, *X’* = *UΣV*^*T*^, where *U* and *V* are orthonormal left and right singular vectors respectively, and *Σ* is a diagonal matrix containing singular values. If *W* = *U*, *H* can be found by projecting data matrix *X* onto the PCs in *W*, i.e., *H* = *W*^*T*^*X*. The PC scores in *H* are then used for potential classification.

NMF has been widely used within various fields of facial recognition^26^, imaging^31,32^, and time-series^33^. The approach has also been employed for biomolecular spectral decomposition^5,24^. Unlike PCA, NMF only uses nonnegativity constraints and is thus particularly well-positioned to retrieve the individual basis spectra for the key fluorophores^34^. Using this non-negative technique on an inherently positive spectral dataset has been shown to allow for the extraction of intrinsic fluorescence spectra^5,35^. NMF allows for the choice of internal dimensionality (*r*-value) for the solution matrix pair. The best-performing choice in *r*-value has been shown to be problem-specific. The problem that NMF attempts to solve is a multiplicative reconstruction of a given input matrix by minimizing the Frobenius norm, reaching a convergence in the process. This is shown mathematically as *X*^*m*xn^ = *W*^*m*x*r*^ x *H*^*r*x*n*^, with ||*X* − *WH*||_*F*_ < *t*, where *t* notates some threshold value. To find *W* and *H*, the values of *W* and *H* are initialized randomly, and updated using a certain algorithm such as a multiplicative update rule or alternating least squares^26,34^. Thus, non-unique convergence is inherent. Therefore, Tikhonov regularization is commonly used to achieve convergence. Once convergence has been reached, matrix *W* is the resultant “feature matrix” containing *r* characteristic spectra that allow it to re-represent the spectra within the input matrix *X* using various weights contained in matrix *H*. Since the update condition turns every negative value to zero, the obtained matrix *W* has increased sparsity and resultant columns which give interpretable extracted spectral information.

Once the weights (scores) are obtained using either PCA or NMF, support vector machines (SVMs) are used to classify the spectra based on the weights in *H*. SVMs are widely used in classification problems to determine the boundary (commonly referred to as a hyperplane) that results in the largest separation between classes based on the closest data points which are called support vectors (SVs). Both linear and Gaussian radial basis function (RBF) kernels were employed.

To avoid bias in the classification, leave-one-out cross-validation (LOOCV)^36,37^ was used with SVM. LOOCV is a widely used technique for validation of discriminative performance. LOOCV begins by discarding a data point from the set. It then fits an SVM separation boundary using the remaining data and subsequently determines whether the previously left-out data point would be correctly grouped using the newly defined SVM separation. This process is iterated through every data point and provides an overall performance accuracy. This method provides a more robust evaluation of the classifier.

To further investigate the above models including PCA-SVM and NMF-SVM, the optimal number *r*_*opt*_ of the most relevant components for classification, which is considered a hyperparameter, was also evaluated. The process for hyperparameter optimization is similar to feature selection. To find *r*_*opt*_, a nested leave-one-out cross validation (LOOCV) method^37–40^ was used to evaluate this hyperparameter. The internal LOOCV was used to evaluate this parameter. To avoid bias in the decomposition, the decomposition was performed only on the training set in the internal LOOCV to find the component loadings during hyperparameter optimization. The component loadings were then used to find a set of component scores for the test set in the internal LOOCV for validation. The optimal value of the hyperparameter was determined based on the highest accuracy^37^. Once the optimal hyperparameter was found, the final evaluation was then performed using the external LOOCV. Similarly, new component loadings were found using the training set in the external LOOCV and used to find new component scores for the test set in the external LOOCV for final evaluation.

The performance of a two-class classification was evaluated using statistical measures such as sensitivity, specificity and accuracy^41^. A receiver operating characteristic (ROC) curve was used as another method to evaluate the performance of the model. The ROC curve was plotted as Sensitivity vs. 1-Specificity by varying the discrimination threshold for the binary classifier^42^. The area under the ROC curve, referred to as AUROC was used as a statistical measure to describe the predictive performance of the model^43,44^. For multi-class classification with more than two classes involved, accuracy for classifying each class and an overall accuracy were used to evaluate the performance of the classification.

## Experimental results

The viability of all the three cell samples used in this study were ~ 98% before spectral measurements using the cells in a quartz cuvette. This study was focused on the efficacy of the technique. Therefore, we require the optical signal be reliable and not change significantly during the signal integration. This was confirmed in the repeated measurements (data not shown here).

**Figure 1:**
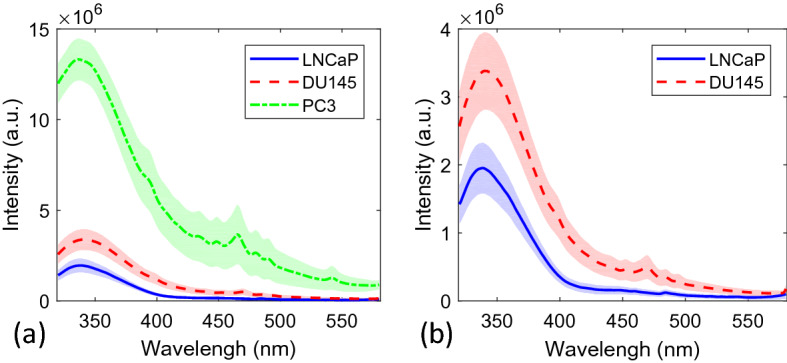
Average and error bounds of raw NFL spectra from (**a**) all three cell lines and (**b**) DU145 and PC-3.

A total of 24, 17 and 23 NFL spectra were collected from the LNCaP, DU145 and PC-3 cell samples respectively by using 300 nm excitation. The average spectra of these acquisitions with error bounds are shown in Fig. 1a. For all three groups, the strongest fluorescence peak is located at ~ 340 nm, which is the characteristic emission peak of tryptophan. Because of high fluorescence intensity from PC-3 much stronger than the other two samples, a second plot is given in Fig. 1b with PC-3 removed to provide a higher clarity for the other two cell types. As observed in these two plots, a peak at ~ 465 nm is prominent in the DU145 spectra as well as in PC-3, which is considered to mainly contributed by NADH. It should be noted that across all wavelengths, the average fluorescence intensity collected from LNCaP is observed to be of lowest value, followed by DU145 and PC-3.

PCA and NMF were then utilized to determine the effectiveness of NFL for distinguishing metastatic potentials among different prostate cells. A 2D SVM boundary was first tested based on the first two components to classify between the LNCaP group and the other two (lowest risk vs. two highest) groups as well as between the PC-3 group and the remaining groups (highest risk vs. two lowest).

**Figure 2:**
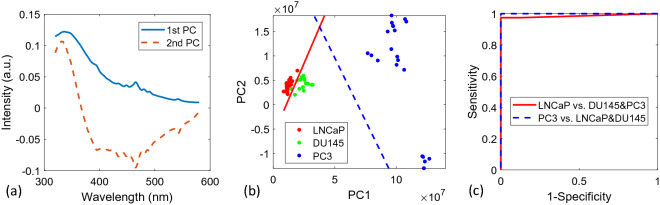
(**a**) Overlay of the first two extracted PCs. (**b**) Scatterplot of the first two PC scores for each NFL spectrum. The solid line separates the least advanced cell line (LNCaP) from the more advanced (DU145 and PC-3) and the dashed line shows the SVM boundary between the cell line possessing highest aggression (PC-3) and the two lowest (LNCaP and DU145). (**c**) The corresponding ROC curves for classifications with LOOCV.

The first two basis spectra retrieved by PCA and a scatter plot of the weights with trained SVM classifiers are shown in Fig. 2a,b, respectively. The variances for the first two PCs contribute 99.97% of the total variance.

**Figure 3:**
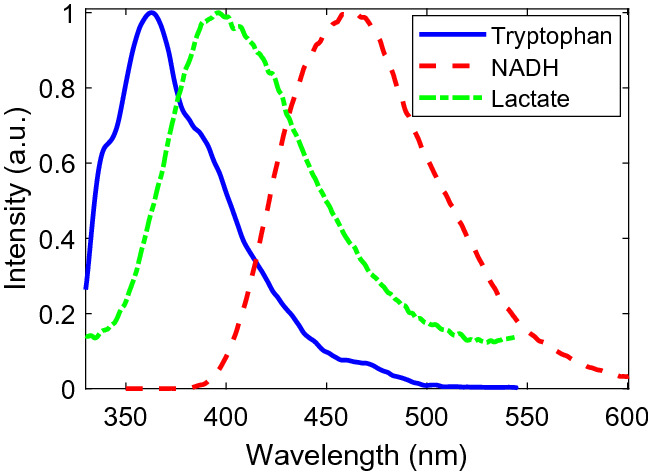
Fluorescence spectra of aqueous solutions of the three fluorophores.

Figure 2a illustrates that the first two PCs are mainly attributed to tryptophan (~ 340 nm) and NADH (~ 465 nm) fluorescent signals. As illustrated by Fig. 2b, classification using these PCs, especially the first PC, provides strong evidence for their effectivity in prostate cell line discrimination. Since PCA allows for the existence of negative values, the 2nd PC possesses both a positive tryptophan peak and a broad negative peak above ~ 370 nm. The broad negative peak contains a peak about 460 nm which is attributed to NADH, along with a shoulder at ~ 400 nm which may be attributed to lactate^45^. More insightful understanding needs be obtained to confirm the source of this peak. Although the spectra of the fluorophores in cells are not expected to be exactly the same as those for the corresponding pure chemicals, we collected and show the fluorescence spectra of the aqueous solution of the chemicals in Fig. 3 below for comparison with the retrieved component spectra. The spectra in Fig. 3 are normalized to the spectra maxima, and consistent with literature data with emission maxima shown around ~ 360 nm, ~ 395 nm, ~ 460 nm for the three chemicals of tryptophan, lactate and NADH, respectively.

The SVM classifier was further evaluated with LOOCV. The corresponding ROC curves are shown in Fig. 2c. The resulting values of sensitivity, specificity, accuracy and AUROC are shown in Table 1.

**Table 1:** Cross-validated sensitivity, specificity, accuracy and AUROC for LOOCV SVM using PCA.

(PCA) Classifiers	Sensitivity (%)	Specificity (%)	Accuracy (%)	AUROC
Least aggressive vs. rest (LNCaP vs. DU145&PC-3)	97.4	100.0	98.4	0.986
Most aggressive vs. rest (PC-3 vs. LNCaP&DU145)	100.0	100.0	100.0	1.0

**Figure 4:**
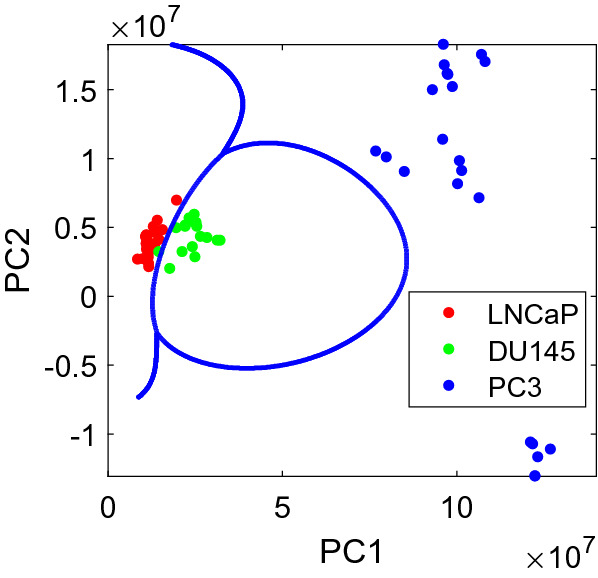
A multi-class SVM classification.

A multi-class SVM classification was also performed for PC1 and PC2 using a Gaussian kernel^25^. The classifier trained using all data is shown in Fig. 4. The LOOCV sensitivity, specificity, and accuracy were found to be 100.0%, 94.1%, and 100.0%, along with a total accuracy of 98.4%.

To further evaluate the model, the optimal number of PCs, *r*_*opt*_, as a hyperparameter was evaluated using multi-class classification by SVM and a nested LOOCV. The *r*_*opt*_ value for PCA-SVM was found to be 3 based on the maximum accuracy. The external LOOCV was used for a final evaluation. The classification accuracies for all the cell types were found to be 95.8%, 100%, and 100% for LNCaP, DU145 and PC-3 respectively. A total accuracy was found to be 98.4%.

**Figure 5:**
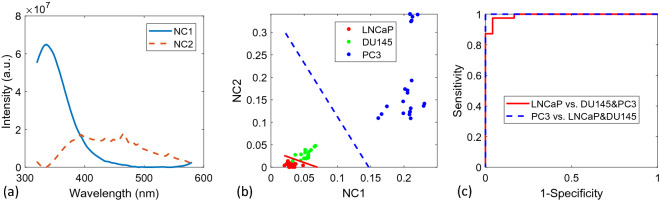
(**a**) Overlay of the first two extracted NMF feature spectra (solid and dashed corresponding to 1st and 2nd respectively). (**b**) Scatterplot of the corresponding NMF-related weight values for each NFL spectrum. The solid line separates the least advanced cell line (LNCaP) from the more advanced (DU145 and PC-3) and the dashed line shows the SVM boundary between the cell line possessing highest aggression (PC-3) and the two lowest (LNCaP and DU145). (**c**) The corresponding ROC curves for classification with LOOCV.

The other multivariate analysis method tested in this study was NMF. An overlay plot of the first two NMF-extracted nonnegative components (NCs) i.e., feature spectra is shown in Fig. 5a. In the initial analysis, *r*-value of 2 was used. As shown in Fig. 2a, the 1st NMF spectral feature shows a strong similarity with the fluorescent emission profile of tryptophan^10^. The NADH peak about 460 nm is not prominent. The 2nd NMF feature spectrum shows a NADH peak along with a peak at ~ 400 nm, which again might be due to lactate. Due to the inherent non-negativity of NFL spectra, the resulting feature spectra obtained by utilizing the positively-constrained technique, NMF, are more easily interpretable than with PCA. Figure 5b indicates both the relative concentrations of tryptophan and NADH and/or lactate (NADH/ lactate) increasing with the aggressiveness of the cancer cells.

Table 2 illustrates the LOOCV sensitivity, specificity and overall accuracy based on the two NCs. The LOOCV ROC curves are shown in Fig. 5c.

**Table 2:** Cross-validated sensitivity, specificity, accuracy and AUROC for LOOCV SVM using NMF.

(NMF) Classifiers	Sensitivity (%)	Specificity (%)	Accuracy (%)	AUROC
Least aggressive vs. rest (LNCaP vs. DU145&PC-3)	97.4	100.0	98.4	0.992
Most aggressive vs. rest (PC-3 vs. LNCaP&DU145)	100.0	100.0	100.0	1.0

**Figure 6:**
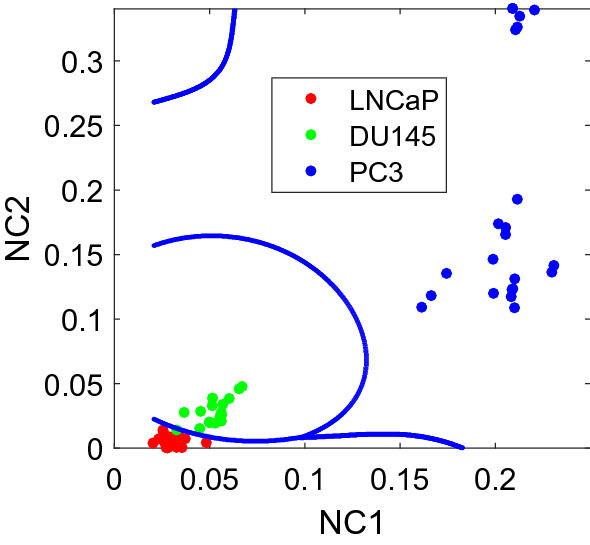
A multi-class SVM classification.

Similarly, a multi-class SVM classification was also performed with a Gaussian kernel using NC1 and NC2, as shown in Fig. 6. The LOOCV sensitivity, specificity, and accuracy were found to be 100.0%, 94.1%, and 100.0%, along with a total accuracy of 98.4%.

The optimal number of NCs, *r*_*opt*_ was also evaluated using multi-class classification by SVM with a nested LOOCV. For simplicity, for any *r*-value used in NMF, all the *r* NCs will be then used for classification. The *r*_*opt*_ value for NMF-SVM was found to be 2. The classification accuracies based on the final evaluation in the external LOOCV were found to be identical to those above based on NMF without cross validation, i.e., 100.0%, 94.1%, 100.0%, and 98.4% for LNCaP, DU145, PC-3 and overall accuracy respectively.

The multi-class SVM classification with LOOCV for the two models based on PCA and NMF are summarized in Table 3 for comparison, where “PCA, SVM-LOOCV” means PCA was performed on the whole dataset and LOOCV was only used with SVM, while “PCA&SVM-LOOCV” means both PCA and SVM were performed on the training set in the cross-validation loops, as explained in detail above. Same is true for the classifier labels with NMF in the table.

**Table 3:** Cross-validated sensitivity and specificity values obtained from NFL classification using NMF.

Classifiers	Accuracy (%) (LNCaP)	Accuracy (%) (DU145)	Accuracy (%) (PC-3)	Accuracy (%) Overall
PCA, SVM-LOOCV	100.0	94.1	100.0	98.4
PCA&SVM-LOOCV	95.8	100.0	100.0	98.4
NMF, SVM-LOOCV	100.0	94.1	100.0	98.4
NMF&SVM-LOOCV	100.0	94.1	100.0	98.4

**Figure 7:**
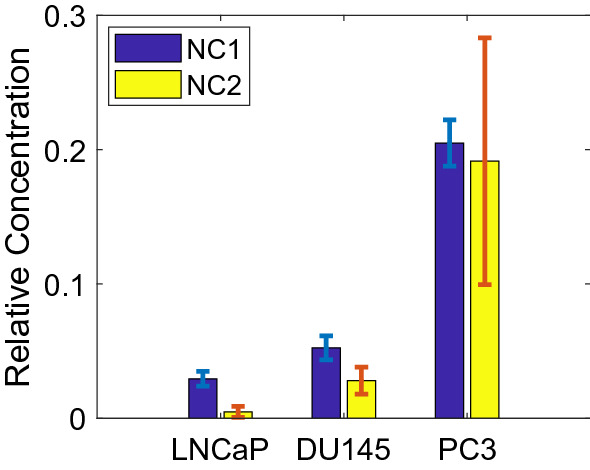
Relative concentrations of tryptophan and NADH/Lactate in three types of cells based on NC scores.

Table 3 shows the performances of the classification of cells with different metastatic potentials based on these two methods are almost identical. But the NCs retrieved by NMF are better interpretable. The relative concentrations of tryptophan and NADH/lactate indicated by NC1 and NC2 are plotted in Fig. 7, which clearly shows an increase in both components with the aggressiveness of the cancer cells. It is evident that errors also increase with the relative concentrations of the NCs across the cell lines. We hypothesize that this may be attributed to the increased inhomogeneity with increasing malignancy or aggressiveness of the cancer cells.

## Summary and discussion

In this study, PC-3, DU145 and LNCaP cell lines were investigated using native fluorescence spectroscopy. Though all three cell lines used are prostate cancer cells, they are different cell lines with differences in biological characteristics^27,28^. LNCaP cells are androgen-sensitive human prostate adenocarcinoma cells. DU145 cells are not hormone-sensitive and do not express prostate-specific antigen (PSA). PC-3 cells do not respond to androgens either, as well as to glucocorticoids or fibroblast growth factors.

All data were collected under the same experimental conditions and the raw data was analyzed to distinguish the cell types without normalization. It was assumed that the absolute signal levels directly reflected chemical concentrations. However, what we presented in the paper was still relative concentrations of the fluorophores among samples. We did not directly compare fluorescence signals between cells and homogeneous solutions to retrieve absolute molar concentrations of the fluorophores. The local environment of the fluorophores is not the same between live cells and homogeneous solutions, and the fluorescence properties of the molecules may not be the same. Therefore, usually they cannot be compared directly to retrieve the absolute concentrations of the fluorophores^46^. This will be further verified during future investigation.

The spectral data were analyzed using machine learning based analysis methods PCA-SVM and NMF-SVM. In particular, multivariate analysis methods PCA and NMF were used to unmix the signal, reduce the data dimension, and detect spectral features. SVMs were used to classify different types of cell lines. This study provided further evidence for the potential of the NFL technique to discriminate among cancerous prostate cells of different metastatic ability with high accuracy. Classification between the most aggressive group (PC-3) with the other groups (LNCaP and DU145) was perfect for all methods illustrated herein. Results also showed that the accuracy for distinguishing the three different types of the cell samples using the two analytic methods was almost identical. A large sample size may provide a better comparison between the methods. But the two basis spectra and the coefficients obtained by NMF are more interpretable, and may be interpreted as the estimated fluorescence spectra of cell intrinsic biomolecules such as tryptophan, NADH and probably lactate, and their corresponding relative concentrations. Indeed, the relative concentrations of these biochemicals in PC-3 seemed to be much higher than those of DU145 and LNCaP, with the latter two being observed as significantly closer to each other (Fig. 7).

In this study, using a multi-class (three-class) classification, we found classifiers to separate every class from other two classes. The data and results of this study showed a direct relationship between the metastatic potential of the prostate cancer cells and the relative concentrations of the key biomolecules such as tryptophan and NADH retrieved from the NFL spectra.

The androgen receptor (AR) plays a critical role in the metastasis of prostate cancer cells, but its mechanism remains unknown^47^. LNCaP cells are androgen-sensitive, while DU145 and PC-3 cells are not^28^. But based on NFL spectral analysis, the difference between LNCaP and DU145 is small, while the difference between PC-3 and the combined group of LNCaP and DU145 is much larger, as evident in Figs. 3, 4, 5, and 6. Therefore, the classification of metastatic ability of the cells in our results cannot be entirely explained by hormone sensitiveness. Even though understanding the mechanism of metastatic potential of prostate cancer cells are related to this study, the main purpose of this paper is to report a technique that may be used to detect cells with different metastatic potential.

Tryptophan is the main source of fluorescence signal in tissues and cells when excited at ~ 300 nm^35,48,49^. The emission peak of tryptophan in aqueous solution was shown to be at about ~ 350 nm^50,51^. This is consistent with our own observation in Fig. 3. Protein bound tryptophan has also been extensively studied in the literature^52,53^. The emission peak wavelength of tryptophan in protein is sensitive to its local environment and ranges from ~ 308to ~ 355 nm^53,54^. In the results of this study, the peak assigned to NADH is usually mixed with another peak (shoulder) around ~ 400 nm, which we proposed to assign to lactate. Lactate is another key molecule that is involved in carcinogenesis^55,56^. Due to the Warburg effect, which is considered to be a hallmark of cancer, cancer cells show high rates of glycolysis in the hypoxia condition or even with oxygen^55,56^. Higher rates of glucose metabolism through anaerobic glycolysis leads to a higher rate of L-Lactate release in cancer cells compared to normal cells^57,58^.

In this study, the relative concentrations of tryptophan and NADH/lactate were found to correlate with the metastatic ability of the cancer cells. They may provide the criteria for prediction of tumor metastasis. Further, the combination of NFL spectroscopy and machine learning based analysis illustrates a high degree of capability for evaluation of tumor metastasis. NFL has the potential to be an alternative optical tool for medical armamentarium^59^. Compared to other techniques for detection of metastatic potential of cancer cells such as those by Paidi et al.^60^ and Bendau et al.^61^, the NFL technique can not only be used for measurements with cells in vitro and fresh tissue specimens ex vivo, but also for in vivo measurements. Since it does not involve spatial scanning (mapping), it works faster than those that require mapping^60,61^, and can potentially be implemented for real-time diagnosis during surgery.

Since we only used three cell lines, whether or not the above-mentioned correlation between the relative concentrations of the key fluorophores and the metastatic potential of the cell lines universally exists for other prostate cell lines needs to be further verified. Future studies will continue to collect more data using different cell lines, obtain more insightful understanding of the spectra, and further test the fluorescence spectroscopic measurements using other excitation wavelengths.

## Data Availability

The data that support the findings of this study are available from the corresponding author upon reasonable request.
